# Supplementary material for: Assessing Genetic Variation among Strychnos spinosa Lam. Morphotypes Using Simple Sequence Repeat Markers
Source: Plants (Basel). 2023 Jul 28;12(15):2810. doi: 10.3390/plants12152810 (PMC10421500; doi:10.3390/plants12152810)
Supplement: Supplementary file 1 [file plants-12-02810-s001.zip › plants-2518795-supplementary.pdf]

plants MDPI

Table S1. Nei's genetic distance among *Strychnos spinosa* morphotypes using fourteen simple sequence repeat markers.

Morphotypes	GRxCR-dGEO	GSR-GEF	GvRR-GRO	PRR-dGEF	GRP-GEF	GSR-dGRF	GRP-GEO	GSR-GEO	GRP-dGRO	GRR-dGEO	GRR-dGRO
GSR-GEF	1.11										
GvRR-GRO	0.96	0.91									
PRR-dGEF	0.76	0.86	0.76								
GRP-GEF	0.91	0.91	0.96	0.71							
GSR-dGRF	1.06	0.76	0.91	0.86	0.96						
GRP-GEO	0.91	0.61	1.06	0.91	0.91	0.71					
GSR-GEO	0.76	0.96	1.01	1.01	0.96	0.81	0.81				
GRP-dGRO	1.01	0.96	0.96	0.91	0.86	0.56	0.86	0.86			
GRR-dGEO	0.96	1.06	0.76	0.71	0.86	0.86	0.91	1.06	0.86		
GRR-dGRO	1.06	0.96	1.11	0.91	1.06	0.76	0.76	0.96	0.66	0.71	
GRR-GEO	0.56	0.96	0.91	0.96	0.86	1.01	0.91	1.01	0.81	1.01	1.01
GRR-GRO	0.96	1.01	1.21	0.96	0.91	1.06	0.91	1.21	1.11	1.06	0.76
GRxCP-GEF	0.96	1.06	1.01	0.86	0.86	1.01	1.06	1.11	1.01	0.71	0.86
GRP-dGEF	0.91	0.96	0.71	0.81	0.81	0.86	0.86	0.76	0.66	0.81	0.96
GRR-GEF	0.86	1.16	1.01	0.86	0.86	0.76	0.91	1.01	0.61	0.81	0.86
PRR-dGRF	0.76	1.01	1.01	0.76	1.01	1.06	0.96	0.76	0.76	1.16	0.96
GvRxCR-GEF	1.01	0.91	0.61	0.66	0.91	0.71	0.91	0.91	0.81	0.91	0.96
PRxCP-GEO	0.81	1.11	0.96	0.76	0.61	0.91	0.81	1.06	0.96	0.71	1.01
GRP-dGEO	1.06	0.91	0.81	0.81	1.06	0.81	0.66	0.86	0.81	0.76	0.91
GRxCR-dGRO	1.11	1.01	0.91	0.91	0.81	1.01	1.01	1.21	0.91	0.86	1.01
GRxCR-dGEF	0.91	1.16	1.06	1.26	1.21	1.21	1.06	1.01	1.11	1.01	1.26
GSxCR-dGRF	1.06	0.66	0.81	0.66	0.66	0.76	0.71	1.01	0.71	0.71	0.81
GRxCR-GEF	0.91	1.11	0.86	0.81	1.11	0.91	0.96	0.71	0.86	0.96	1.01
GSR-GRO	0.91	0.71	1.16	0.86	0.91	0.81	0.10	0.91	0.86	0.91	0.76
GvRR-dGEO	0.66	1.16	0.91	0.76	0.96	0.81	0.91	0.91	0.66	0.81	0.91
GRxCP-dGEF	1.16	1.01	1.01	0.86	0.96	0.91	0.76	0.96	0.81	0.76	0.81
GRP-GRO	0.86	1.16	1.01	0.86	0.96	0.91	0.76	1.06	0.86	0.91	0.96
GvRR-dGRO	0.96	0.76	0.81	0.81	0.81	0.76	0.66	0.86	0.66	0.86	0.81
GRxCP-GEO	0.86	0.71	1.01	0.76	0.91	1.11	0.86	0.86	1.11	0.86	0.91
PRxCP-dGEO	0.86	0.96	1.16	0.86	1.06	0.76	0.76	0.66	0.76	1.06	0.91

PRR-dGRO	0.91	0.91	0.86	0.86	0.91	0.81	0.71	0.96	0.71	0.81	0.81

GSR-GEF										
GvRR-GRO										
PRR-dGEF										
GRP-GEF										
GSR-dGRF										
GRP-GEO										
GSR-GEO										
GRP-dGRO										
GRR-dGEO										
GRR-dGRO										
GRR-GEO										
GRR-GRO	1.01									
GRxCP-GEF	0.96	1.06								
GRP-dGEF	0.81	1.06	0.96							
GRR-GEF	0.91	0.91	0.91	0.66						
PRR-dGRF	0.81	0.96	1.16	0.76	0.81					
GvRxCR-GEF	0.96	1.06	1.06	0.71	0.81	1.01				
PRxCP-GEO	1.11	0.96	0.91	0.96	0.86	0.96	0.91			
GRP-dGEO	1.11	1.21	0.96	0.71	1.01	0.86	0.66	0.81		
GRxCR-dGRO	0.91	1.11	0.86	0.56	0.86	1.11	0.81	1.01	0.86	
GRxCR-dGEF	0.91	1.21	1.26	1.16	1.11	1.11	1.16	1.01	1.16	1.21
GSxCR-dGRF	0.96	0.86	0.86	0.56	0.81	0.81	0.71	0.81	0.71	0.81
GRxCR-GEF	1.26	1.11	1.21	0.86	0.86	0.86	0.71	0.76	0.76	1.11
GSR-GRO	0.91	0.91	1.06	0.86	0.91	0.86	1.01	0.81	0.76	1.01
GvRR-dGEO	0.96	1.01	1.06	0.76	0.30	0.66	0.71	0.76	0.91	0.96
GRxCP-dGEF	1.11	1.21	0.76	0.61	0.86	0.86	0.86	0.81	0.20	0.76
GRP-GRO	0.81	0.96	0.91	0.66	0.66	0.91	1.01	0.71	0.86	0.91
GvRR-dGRO	0.86	0.91	0.86	0.76	0.81	0.91	0.81	0.91	0.71	0.91
GRxCP-GEO	0.81	1.06	0.91	1.06	1.21	1.01	1.11	0.96	1.01	1.11
PRxCP-dGEO	1.06	1.06	1.11	0.96	0.71	0.76	0.91	0.96	0.96	1.11
PRR-dGRO	0.71	1.11	0.76	0.76	0.86	0.96	0.81	0.81	0.66	0.86

GSR-GEF	
GvRR-GRO	
PRR-dGEF	

GRP-GEF										
GSR-dGRF										
GRP-GEO										
GSR-GEO										
GRP-dGRO										
GRR-dGEO										
GRR-dGRO										
GRR-GEO										
GRR-GRO										
GRxCP-GEF										
GRP-dGEF										
GRR-GEF										
PRR-dGRF										
GvRxCR-GEF										
PRxCP-GEO										
GRP-dGEO										
GRxCR-dGRO										
GRxCR-dGEF										
GSxCR-dGRF	1.21									
GRxCR-GEF	1.11	0.96								
GSR-GRO	1.06	0.71	1.06							
GvRR-dGEO	0.91	0.91	0.66	0.81						
GRxCP-dGEF	1.26	0.61	0.96	0.76	1.01					
GRP-GRO	1.11	0.91	1.06	0.71	0.76	0.76				
GvRR-dGRO	1.06	0.71	0.91	0.76	0.81	0.76	0.81			
GRxCP-GEO	1.21	0.81	1.26	0.81	1.16	1.01	1.06	0.86		
PRxCP-dGEO	1.11	1.06	0.81	0.86	0.61	1.06	0.91	0.76	1.11	
PRR-dGRO	1.06	0.86	0.91	0.81	0.91	0.66	0.71	0.61	0.96	0.86

Morphotypes are explained in Table 2
